# The Design of Abnormal Microenvironment Responsive MRI Nanoprobe and Its Application

**DOI:** 10.3390/ijms22105147

**Published:** 2021-05-13

**Authors:** Ancong Wang, Xiao Han, Wenliu Qi, Sihui Du, Zhenqi Jiang, Xiaoying Tang

**Affiliations:** School of Life Science, Institute of Engineering Medicine, Beijing Institute of Technology, Beijing 100081, China; awang@bit.edu.cn (A.W.); 3120170646@bit.edu.cn (X.H.); 3220201324@bit.edu.cn (W.Q.); 3220181071@bit.edu.cn (S.D.)

**Keywords:** MRI nanoprobe, environment responsive, design, application

## Abstract

Magnetic resonance imaging (MRI) is often used to diagnose diseases due to its high spatial, temporal and soft tissue resolution. Frequently, probes or contrast agents are used to enhance the contrast in MRI to improve diagnostic accuracy. With the development of molecular imaging techniques, molecular MRI can be used to obtain 3D anatomical structure, physiology, pathology, and other relevant information regarding the lesion, which can provide an important reference for the accurate diagnosis and treatment of the disease in the early stages. Among existing contrast agents, smart or activatable nanoprobes can respond to selective stimuli, such as proving the presence of acidic pH, active enzymes, or reducing environments. The recently developed environment-responsive or smart MRI nanoprobes can specifically target cells based on differences in the cellular environment and improve the contrast between diseased tissues and normal tissues. Here, we review the design and application of these environment-responsive MRI nanoprobes.

## 1. Introduction

Biomedical imaging is an important diagnostic tool in medical treatment. The most commonly used clinical imaging modalities are: Magnetic Resonance Imaging (MRI), X-ray, Computed Tomography (CT), Ultrasound imaging (US), Positron Emission Tomography (PET), Single Photon Emission Computed Tomography (SPECT). Among them, PET and SPECT involve radioactive materials and only provide physiological information; US technology is widely used in clinical research in obstetrics, cardiology, and surgical guidance, but the effect of detailed observation of deep tissues is not ideal; X-ray is an important tool for clinics since they are cost-effective, fast, and high resolution and have no depth limits, but there are radiation problems; CT has no depth limit and can provide anatomical and physiological information and it is often used for bone disease diagnosis, tumor location, image-guided surgery, and radiotherapy diagnosis [1,2]. Magnetic resonance imaging (MRI) offers high spatial resolution (25–100µm) and soft tissue resolution, and is used a non-invasive monitoring method for the morphological and functional imaging of nerves, heart, liver, etc. to diagnose and evaluate the developmental stage of a disease. MRI relies on the spin-lattice relaxation time (T_1_) or spin–spin relaxation time (T_2_) of protons in different microstructures to produce contrast in the images. The traditional T_1_ and T_2_ single-mode imaging has some limitations, i.e., approximately 35% of clinical MRIs require contrast agents for better sensitivity and diagnostic accuracy, such as for the diagnosis and detection of small tumors in normal tissues. Thus, it is necessary to develop new MRI contrast agents to provide more information regarding the lesion location as well as a high signal-to-noise ratio (SNR) to supplement the traditional imaging data [3].

Molecular imaging uses molecular probes for specific biological targets in the tumor microenvironment to obtain better 3D images regarding the anatomical structure, physiology, pathology, etc., which can provide an important reference basis for early and accurate tumor diagnosis and treatment. In the tumor microenvironment, metabolic, immune and endocrine changes affect the occurrence and development of tumors. Researchers have synthesized targeted molecular probes for specific and highly expressed receptors on neovascular endothelial cells and tumor cells to investigate the occurrence and development of tumors and their structural characteristics at the microscopic level [4]. This targeting molecular probe contains three parts: targeting ligand, transporter, and contrast agent, and it should have an amplifying effect, strong penetrating ability, long half-life, and rapid elimination from the body [5]. The target molecule of the probe should have high secretion or high expression, high affinity, and can well represent the biological characteristics of the target. MR molecular imaging uses tissue-specific expression products as the imaging targets, provides image contrast using non-aqueous molecules, and uses MR molecular probes to understand the physiological and pathological processes at the cellular and molecular level to qualitatively or quantitatively investigate the gene expression, biological metabolism, etc. This enables a comprehensive biological and imaging diagnostic analysis for disease diagnosis, treatment, and relevant basic research [6].

Nanomaterials have high specific surface area, high surface energy and a unique blend of mechanical, thermal, electrical, magnetic, and optical properties. Their physical and chemical properties can be adjusted by changing the composition, shape, size, structure, as well as via surface modification. Nanoparticles (NPs) play an important role in the medical field by virtue of their unique nanoscale characteristics and their core or surface-modified functional groups. By introducing multiple modes into NPs, they can effectively act as imaging contrast agents and can provide complementary information for accurate cancer diagnosis. Most traditional anti-cancer drugs cannot distinguish between normal cells and cancer cells; however, NPs can preferentially accumulate in the tumor area via the enhanced penetration and retention effects (EPR), and can effectively carry and transport imaging probes, therapeutic agents, or biological materials to specific locations, such as specific organs, tissues, and even cells [7]. The composition of the tumor microenvironment is quite different from that of normal tissues. Efficient and selective nanoprobes can help in realizing real-time in situ cell imaging and enable non-invasive cancer treatment in response to tumor microenvironment (hypoxia, slightly acidic, redox, enzyme) or external field stimulation response (light, heat, and magnetism). In recent years, there has been a rapid development of environment-responsive MRI probes. They have stimulus response capabilities and can specifically target cells based on the different characteristics of the cell environment. This significantly increases the cumulative release rate of nanomolecular imaging probes, improves the in vivo imaging efficiency and drug availability, and improves the tracer SNR between the diseased sites and normal tissues [8].

## 2. The Design of the MRI Nanoprobe

^1^H-MRI is often used for imaging analysis during the clinical screening of diseased tissues. It based on the difference in the content of hydrogen atoms in the body and the difference in their relaxation time, allowing for a real-time visualization of the physiological and anatomical features. However, since almost 70% of the human body is made of water, there is only a minor difference in normal and diseased tissues. Additionally, background interference has been shown to cause problems, such as missed diagnosis and misdiagnosis in clinical application. After ^1^H, ^19^F is the preferred nucleus for MRI imaging, which has a natural abundance of 100%, no radioactivity, a gyromagnetic ratio of 94% of that of ^1^H, and sensitivity of 83% of that of ^1^H [9]. Since the human body contains very little fluorine, ^19^F-MRI is almost free from background signal interference when used for in vivo research. In recent years, there has been an increase in the use of environment-responsive MR nanomolecular imaging probes for the detection of changes in pH, enzyme activity, microenvironment, hypoxia, and photothermal [10]. Thus, the development of multi-modal, multi-stimulation environment-responsive MRI nanomolecular imaging probes would play an important role in promoting the development of molecular imaging and the integration of diagnosis and treatment. Further, metal ion such as Zn^2+^, Ca^2+^, Cu^1+^, and Cu^2+^ responsive contrast agents also involved in a number of vital cellular processes and therefore can play a role in the responsive MRI, but limited research has been reported in this field [11,12,13].

There are two types of MRI imaging materials: paramagnetic materials and superparamagnetic materials. Paramagnetic materials mainly include gadolinium (Gd) [14] and manganese (Mn) [15], whereas superparamagnetic materials mainly include iron oxide (IO). Gadolinium ion (Gd^3+^) has seven unpaired electrons and its chelates produce a T_1_-positive contrast effect by changing the magnetic properties of the surrounding hydrogen nuclei. In recent years, researchers have combined Gd-DTPA or Gd-DOTA with antibodies and proteins to develop various macromolecular chelates for targeted molecular imaging research [16,17]; however, long-term retention of Gd in the body might cause nephrogenic systemic fibrosis. Manganese ion (Mn^2+^) is a type of T_1_ contrast agent because it contains five unpaired electrons [18,19]. The nanoprobe prepared by combining Mn^2+^ with NPs has been shown to possess good stability, easy chemical modification, good biocompatibility, and can be used to target drug delivery. However, a high concentration of the manganese chelate can cause irreversible damage to the nervous system, such as the basal ganglia and substantia nigra of the brain. Therefore, it is necessary to further optimize the preparation parameters and biological characteristics of the nanoprobe to reduce clinical toxicity and side effects.

Studies have shown that the superparamagnetic material composed of iron oxide crystals and hydrophilic surface coating can produce a T_2_-negative contrast [20,21]. Thus, nano-sized iron oxide particles have been widely used in molecular imaging research of tumors, stem cell therapy, and cell apoptosis [22]. However, some of the disadvantages of iron-based MR molecular probes include complex surface modification, irregular particle shape, and difficulty in controlling the preparation parameters. New type of superparamagnetic nanoprobes, mostly based on the original super-small particle of iron oxide nanparticles (SPIONs), are targeted to bind ligands on the surface of nanoparticles, such as proteins, peptides, small molecules (such as folic acid, carbohydrates), etc., it specifically binds to tissue cell receptors in the body to assist tumor diagnosis at the microscopic level. In recent years, some scholars have developed a nanoprobe (Fe_3_O_4_-BSAGd) with T_1_-T_2_ dual-mode MRI performance, which realizes a two-way accurate diagnosis of lesions and promotes the development of molecular imaging.

### 2.1. pH-Responsive MRI Nanoprobes

Tumor tissues grow and metabolize rapidly by consuming large amounts of glucose and oxygen, resulting in the accumulation of excessive lactic acid and hydrogen ions in the extracellular fluid. This makes the tumor microenvironment to be weakly acidic (pH 5.5 to 6.8) [23,24]. Thus, the pH-responsive MRI probes can be designed in the following two ways: (1) the probe cleaves and releases ions automatically in response to changes in the environmental pH; (2) the probe changes its shape in response to changes in the environmental pH changes, such as becoming smaller, bigger, or “disintegrate” [25].

The Gd-based probes are usually activated by regulating the accessibility of Gd to water molecules. Different Gd probes are encapsulated in polymer matrix or dendrimers, and they undergo changes in morphology, hydrolysis or protonation when exposed to acidic pH, thereby changing the relaxation value. In some cases, due to the changes in size, the probes would cumulatively cause contrast enhancement. In a mild acid environment, Mn-based oxides have better biocompatibility and less toxicity than Gd-based probes. The rapid rupture of 2D MnO_2_ nanosheets in acid medium has been shown to greatly improve the T_1_ contrast of tumor tissues, which promotes the on-demand release of anticancer drugs, avoiding the drug resistance of cancer cells.

Nishiyama and Kataoka et al. used Mn^2+^ to encapsulate calcium phosphate (CaP) NPs, obtaining PEGMnCaP, which when exposed to an acidic pH environment, could release Mn^2+^ ions combined with proteins, increasing the T_1_ contrast [26]. Zhao et al. designed a smart nanoprobe that could be used as both an imaging agent and a therapeutic drug. They loaded arsenic trioxide (ATO) on silica NPs; the acidic medium triggered the simultaneous release of the clinical anticancer drug ATO and Mn^2^^+^, enhancing the contrast of T_1_ [27]. Li et al. encapsulated MnO NPs in hybrid silica nanoshells and performed bimodal imaging using fluorophores. In an acidic environment, Mn^2+^ was released, enhancing the T_1_ contrast [28]. The researchers also used mesoporous manganese silicate-coated silica NPs for similar studies and found that at pH < 7, the manganese silicate shell release Mn^2+^. Additionally, they also use pore-loaded doxorubicin (DOX) in the combined drug delivery and imaging system. When exposed to acidic pH, the electrostatic interaction between DOX and the pore surface resulted in the release of DOX to achieve targeted therapy [29]. Zhang et al. combined the HMCNs and MnO_x_ NPs through the simple and direct chemical redox reaction between the reduce carbonaceous skeleton and the oxidized MnO_4_^−^. The relaxation value of the prepared MnO_x_-HMCNs increased by 52.5 times in a mildly acid solution. Furthermore, the use of special supramolecular π-π stacking between the carbonaceous skeleton of MnOx-HMCNs and aromatic drug molecules was used to build a smart pH-sensitive drug release nano-platform, which showed a unique anti-metastatic effect and high performance in reversing cancer cell multi-drug resistance [30].

At pH < 6.8, the iron oxide NPs encapsulated in micelles underwent decomposition, resulting in the rupturing of the micelles, followed by the release of iron oxide NPs to enhance the T_2_ contrast [31]. The PEGylated nanogel with hydrophobic fluorinated nanoprobe was not hydrated under basic conditions, and its signal was turned off. At pH < 7, the nanogel hydrated and swelled, increasing the mobility of the fluorinated core, causing the signal to turn on. Preslar et al. designed a series of self-assembled fluorinated peptide amphiphiles by changing the content of charged amino acids and the number of fluorine atoms in the hydrophobic segment to achieve the goal of optimizing the ^19^F MRI signal in response to changes in pH. Studies have shown that the increase in pH will cause the supermolecular aggregates formed by certain amphiphiles to transform from low-curvature nano-scale strips to cylindrical nanofibers, thereby enhancing the MRI signal [32].

Chen et al. obtained a new type of nano-pH-triggered probe, which was based on encapsulating fluorine-containing compounds in the pores of mesoporous silica NPs attached with Au NPs. The Au NPs were connected to the pore surface through pH-sensitive covalent bonds. At pH < 6, the hydrazine bond was hydrolyzed and the fluorine nanoprobe was released, activating the ^19^F-MRI signal [33]. Huang et al. used ionizable diblock copolymers to prepare a series of micelles containing ^19^F, and achieved pH-based environmental response through the decomposition of the micelles, achieving qualitative measurement of environmental pH values via ^19^F-MRI [34].

Sherry et al. synthesized a derivative of Gd-DOTA, GdNP-DO3A, in which one of the carboxyl groups was replaced with p-nitrophenol, which was protonated at low pH, allowing water to approach Gd. The relaxation performance decreased with an increase in pH [35]. Guo used 4-trifluoromethylimidazole to partially replace the 2-methylimidazole in the metal organic framework material ZIF-8 to introduce fluorine atoms. Then, the surface was modified with polyethylene glycol silane to obtain the pH-responsive ^19^F-MRI probe, which had a stable structure at physiological pH. However, a gradual decrease in pH caused the dissociation of the nanoprobe, restoring the activity of the fluorine atom and significantly enhancing the ^19^F-MRI signal.

**Figure 1:**
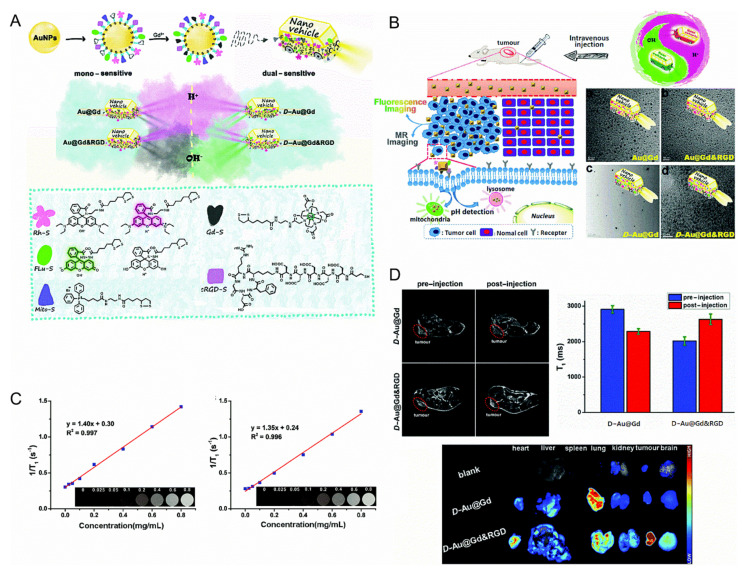
(**A**): Schematic diagram of the nanovehicles. (**B**): Schematic diagram of the nanovehicles and TEM images. (**C**): (Left) in vitro T_1_-weighted MR imaging of D-Au@Gd in PBS, (Right) in vitro T_1_-weighted MR imaging of D-Au@Gd&RGD in PBS. (**D**): (Upper left) the MRI of D-Au@Gd and D-Au@Gd&RGD in U87 tumor-bearing nude mice, (Upper right) the fluorescence imaging, (Lower) the semi-quantitative MRI signal intensity in solid tumors [36]. Reproduced with permission from Royal Society of Chemistry 2020.

Yua et al. designed two pH-sensitive MRI/FI bimodal agents based on AuNPs (Figure 1). The first (Au@Gd and Au@Gd&RGD) contained rhodamine derivatives that were sensitive to acidic conditions, which facilitated the monitoring of the impact of pH changes of lysosomes in living cells. The second (D-Au@Gd and D-Au@Gd&RGD) included two pH-responsive fluorophores, rhodamine and fluorescein derivatives, which could provide a very accurate intracellular pH map and could also be used to calculate the pH of living cells. Additionally, authors also developed an imaging agent (D-Au@GD&RGD) that could determine the location and size of tumors and evaluate the effect of treatment in vivo. It could also be used at the cellular level to monitor the pH changes in cancer cells [36].

Wang et al. developed MRI drug loads and tumor signal amplifiers based on pH-responsive polymers and Gd metal fullerenes (GMF). It had higher relaxation and lower risk of the release of Gd^3+^. At normal pH, GMF and drug molecules were enclosed in the hydrophobic core of the NPs composed of the pH-responsive polymer. Since they were shielded by the aqueous environment, the longitudinal relativity was low, and the drug release was slow. In contrast, in an acidic environment, the MRI signal was amplified, and the drug was released quickly, due to the hydrophobic-hydrophilic transition of the pH-responsive polymer. Thus, this contrast agent had broad prospects in tumor detection and monitoring drug release [37].

### 2.2. Enzyme-Responsive MRI Nanoprobes

Some of the enzymes, such as matrix metalloproteinase, esterase, amylase and cathepsin B, are overexpressed both outside and inside the tumor cells. Caspase-3/7 is a marker of apoptosis. Rao et al. formed Gd NPs that exhibited an r_1_ increasing effect after interaction with caspase-3/7 by regulating self-assembly and macrocyclization. The nanoprobe they synthesized called C-SNAM is composed of a Gd-DOTA complex that is connected to the DEVD peptide, the disulfide bond sensitive to GSH, and the self-assembly of 2-cyano-6-hydroxyquinoline and D-cysteine residues. In the GSH-induced reducing environment inside the cell, cyclization is caused by the degradation of DEVD peptide in the presence of caspase-3/7. The amplification of r_1_ in Gd NPs and the tissue retention due to the increase in size enhances the T_1_ contrast in MRI. They used this nanoprobe to detect caspase-3/7 activity in a chemically sensitive tumor model and successfully completed in vitro and in vivo imaging. Next, they compared it with a nanoprobe that could not be circularized [38]. Coincidentally, after intra-articular injection of matrix-related stem cells, a similar method was used to detect stem cell apoptosis, which could treat cartilage defects [39].

Kikuchi et al. used a short peptide cleaved by caspase-3/7 to link the Gd chelate with a fluorine-containing compound to form a new probe. Due to the paramagnetic relaxation enhancement effect (PRE), the initial ^19^F signal was in the off state, and the paramagnetic compound close to fluorine shortened the T_2_ value, attenuating the MRI signal. When caspase-3/7 cleaved the peptide, the Gd chelate and fluorine separation switched the ^19^F-MRI signal on [40]. In a similar way, Yue et al. used Gd’s PRE effect on fluorine by linking Gd chelate with fluorine using a peptide sensitive to MMP-2 enzyme. They observed that the signal increased when the nanoprobe was exposed to the enzyme [41]. Keliris et al. used β-galactosidase to separate the fluorine nanoprobe from the Gd chelate, thereby eliminating the PRE effect [42]. Yuan et al. designed a smart nanoprobe to visualize the activity of caspase 3/7 in vivo. The fluorine nanoprobe based on DVED peptide was assembled into the NPs in the reducing environment (GSH) in the presence of caspase 3/7. The decomposition of the NPs resulted in the opening of the probe [43].

Liu et al. prospectively designed a fluorinated hydrogel precursor that could successfully detect two enzymes. First, the alkaline phosphatase (ALP) was used to make the hydrogel by the fluorinated precursor, which had an undetectable ^19^F-MRI signal. Second, tyrosine kinase facilitated the regulation of the decomposition of the hydrogel and the consequent activation of ^19^F-MRI signal [44].

Loving and Caravan designed a nanoprobe to detect fresh blood clots based on the activity of protein disulfide isomerase (PDI) to prevent thrombosis. PDI is a biomarker for newly formed blood clots. They chelate the fibrin targeting peptide and the disulfide group that can be modified by PDI for positioning with Gd. When the nanoprobe interacts with PDI, it hydrolyzes the disulfide bond to form a new intramolecular disulfide bond, inducing conformational changes in the peptide, which could bind fibrin, since the abundant fibrin in the blood clot would retain the nanoprobe in the clot. The authors found that r_1_ increased by 70% when the nanoprobe was bonded to fibrin [45].

Cheng et al. used phospholipase (PLA_2_) to degrade glycerophospholipids and tested its activity by loading liposomes with Gd-chelated gadoteridol. When gadoteridol was encapsulated, the permeability of liposomes was reduced, which hindered the accessibility of water molecules, and thus, the T_1_ weighted signal was low. In the presence of PLA_2_, liposomes were degraded and Gd probes were released, leading to significant reduction in the T_1_ relaxation time [46]. Based on the PRE effect, Guo designed ^19^F-MRI nanometers based on the self-assembly of lecithin, polyethylene glycol, Gd^3^^+^-doped upconversion luminescent nanoparticles, and perfluoro-15-crown-5-ether to detect phospholipase (PLA2) activity.

Schellenberger et al. reported that the contrast effect of iron oxide nanocontrast agents based on matrix metalloproteinase-9 (MMP-9) was weaker before the enzyme response. However, after MMP-9 shearing, the iron oxide lost the polyethylene glycol (PEG) that maintained the particle stability, resulting in aggregation that enhanced the T_2_ relaxation effect [47]. Ansari et al. reported an iron oxide NPs probe based on MMP-14 with peptide targeting function. At the tumor site, MMP-14 cleavage of the peptide resulted in the accumulation of probes in the tumor. Thus, compared with the non-targeted probes, its T_2_ contrast was significantly enhanced [48].

Chemical exchange saturation transfer (CEST)-based enzyme detection, such as He used a lanthanide (III) complex as a chemical exchange saturation transfer reagent. Since β-galactosidase cleaved the nanoprobe and turned off the CEST effect, it could be used to detect β-galactosidase activity [49]. Reddy et al. used poly-L-glutamic acid (PLG) as a CEST nanoprobe to detect cathepsin activity in the tumor, causing the difference in CEST signal between cleaved and uncut PLG. This method was supported by both in vitro and in vivo brain tumor models and showed promise as a diagnostic tool in the future.

Pagel et al. used salicylic acid as a catalytic CEST sensor, and fully functionalized salicylic acid as the substrate to detect alkaline phosphatase (AP), sulfa, and esterase. They used phosphaldehyde to detect AP, which had a significant CEST effect before AP was hydrolyzed. By modifying the salicylic acid using the sulfate group in the phenol group and the methyl ester in the acidic part, the presence of sulfonamides and esterase could be detected. Both types of enzymes could hydrolyze the substrate sequentially or simultaneously, reforming salicylic acid, and switching on the CEST signal [50].

### 2.3. Redox-Responsive MRI Nanoprobes

During the growth process, tumor cells produce excessive amounts of reducing substances, much higher than normal tissues. Glutathione protects cells by providing antioxidant defense, controlling cell differentiation and metabolism, etc.

Manganese probes are widely used in the redox response because the Mn^3+^ or Mn^4+^ complexes or salts are stable, easily available and can be reduced to Mn^2+^. The production of Mn^2+^ is often aided by low pH because both low pH and GSH are present in cancer cells. Some pH-triggered manganese probes are considered to be dual-triggered probes of pH and redox [51,52]. Some studies use MnO_2_ nanosheets that can be adsorbed on the surface of drugs or biomolecules. Under reducing conditions, the drugs are released together with Mn^2+^ to enhance the contrast of MRI. This technology has been successfully used for in vitro and in vivo cancer imaging [53,54].

Kim et al. synthesized the T_1_/T_2_ dual-mode nanoprobe using Mn_3_O_4_ to wrap iron oxide NPs. In the closed state, the relaxation rate of the nanoprobe was very low; however, in the presence of GSH, the shell decomposed into Mn^2^^+^, exposing iron oxide NPs and increasing r_1_ and r_2_ [55]. Some researchers used Fe^3+^/Fe^2+^pairs as reversible switches to turn on and off the signal. The researchers prepared a cubic octamer polyhedral oligosiloxane, in which ferrocene was used as a redox switch and trifluoromethyl was used as a fluorine probe. If the iron was present as Fe^2+^, the signal was in the “on” state; however, when ammonium persulfate (APS) oxidized it to Fe^3+^, the signal was turned “off”. A mild reducing agent could reduce the system to Fe^2+^ to turn on the signal again [56].

Muñoz Úbeda combined the Gd chelate with the silica particles using a short, redox linkage composed of disulfide bonds. The presence of GSH caused the cleavage of the bond, releasing the Gd chelate from the silica particles, and significantly increasing r_1_ due to the increase in the roll rate [57]. Nakamura et al. encapsulated fluorine-containing probes in silica NPs, and then connected them to Gd chelate via redox-sensitive disulfide bonds. When the Gd chelate was attached to the surface of silica NPs, the ^19^F-MRI signal was turned off. In a reduced state, the disulfide bonds underwent cleavage and the Gd chelate separated from the probe, thereby turning the ^19^F-MRI signal “on” [58].

Kadakia reported two high-fluorine Cu-based contrast agents, CuL_1_ and CuL_2_, as nanoemulsion preparations to detect cell hypoxia. Since Cu^2+^ is paramagnetic, both these complexes could retain their initial quenched ^19^F-MRI signal. However, when the complex was reduced, the ^19^F-MRI signal increased [59]. Additionally, the nanoprobe reported by Kadakia had a dual response to cellular hypoxia: ^19^F-MRI and fluorescence imaging. Under normal conditions, the copper-based nanoprobe had no ^19^F-MRI signal, and the fluorescence signal was also reduced due to paramagnetic quenching. In a reducing environment, after it was reduced to Cu^+^, the nanoprobe operated in two modes. Additionally, this bimodal nanoprobe could distinguish hypoxic and normoxic cells [60]. Xie et al. introduced a series of fluorinated Cu^2+^ ATSM derivatives, which could be used as ^19^F-MRI agents to detect cell hypoxia. The synthesized complex targeted the hypoxic Cu^2+^ coordination core, and nine equivalent fluorine atoms were connected by variable-length polyethylene glycol. The introduction of the fluorine-based layer maintained the plane coordination geometry of the Cu^2+^ center, while the joint length adjusted the Cu^2+^/Cu^+^ reduction potential, ^19^F-MRI relaxation characteristics, and lipophilicity. The author proved that adjusting the distance between Cu^2+^ and F atoms could enhance ^19^F-MRI relaxation [11].

**Figure 2:**
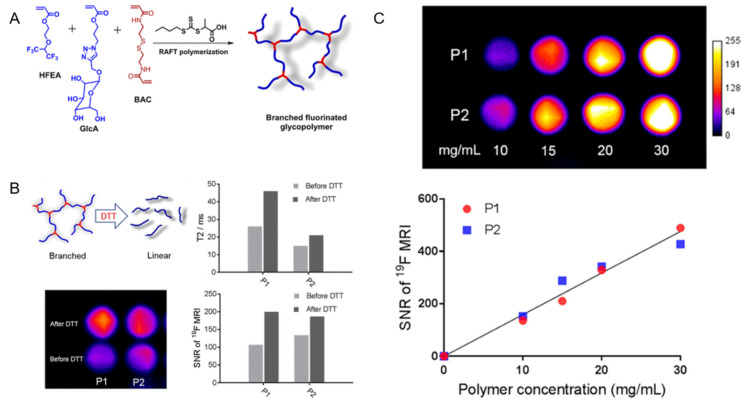
(**A**): Synthesis of branched fluorinated glycopolymers by a one-pot RAFT Polymerization. (**B**): (Upper left) the transformation of a branched structure to a linear structure when the polymer is exposed to high levels of DTT; (Upper right) a comparison of the T_2_ relaxation time of P1 and P2 before and after treatment with 10 mM DTT; (Lower left) a comparison of ^19^F-MRI images of P1 and P2 solutions before and after 10 mM DTT treatment; (Lower right) the signal-to-noise ratio of ^19^F-MRI of P1 and P2 solutions before and after 10 mM DTT treatment. (**C**): (Upper) the ^19^F-MRI of P1 and P2 solutions; (Lower) the ^19^F-MRI signal-to-noise ratio of P1 and P2 solutions within a certain concentration range [61]. Reproduced with permission from American Chemical Society 2019.

Fu et al. reported a new ^19^F-MRI contrast agent, a branched-chain fluorinated glycoprotein, which could target cancer imaging in response to a reducing environment (Figure 2). In the presence of disulfide-containing crosslinking monomers, one-pot RAFT polymerization of glucose and fluoro-monomers could easily prepare fluoro-glycoproteins. Since fluorinated sugar polymers interacted with sugar transporters overexpressed on the cell surface, the binding of glucose units along the polymer chain allowed them to effectively target cancer cells. Additionally, the polymer exhibited enhanced ^19^F-MRI signal under reduced conditions [61].

### 2.4. Other Examples of MRI Nanoprobes

Preslare et al. used nanostructures formed by self-assembly of fluorinated amphiphilic peptides to detect Ca^2+^. The high fluorinated peptide amphiphile self-assembled to form nanobelts in the buffer solution. When the Ca^2+^ concentration in the system increased, the width of the nanobelts increased and consequently the signal intensity of ^19^F-MRI decreased. The fluorine atom-modified lectin biosensor had a sharp ^19^F-MRI peak; however, in the presence of glycoproteins, the sensor’s sharp ^19^F-MRI peak disappeared. The biosensor could also be used to distinguish glycoproteins from small molecule sugars [13].

Deng et al. constructed an MRI nanoprobe Ce6/Fe_3_O_4_-M by co-encapsulating the photosensitizer Ce6 and Fe_3_O_4_ NPs in mPEG2000-TK-C16 micelles. They designed a thioketal (TK) connector in the MRI probe, which was less sensitive to the reactive oxygen species (ROS) in physiological and pathological environments but was highly sensitive to singlet oxygen. When TK was cracked by elemental oxygen generated by light irradiation, it caused the loss of polyethylene glycol on its surface, causing an increase in hydrophobicity and aggregation of Fe_3_O_4_ NPs, resulting in a negatively enhanced T_2_-weighted MRI signal. Thus, this nanoprobe could be used to evaluate the singlet state of photosensitizers [62].

He et al. used hydroxyapatite (HAp) with excellent biocompatibility and good stability in a wide range of pH and temperature, at the same time, they used tetrahedral DNA nanostructures conjugated with AS1411 aptamers (TDN) as an affinity ligand, a new monodisperse HAp-based nanoprobe (Apt-TDNs-GdHAp) doped with Gd^3+^ was constructed for MRI. Furthermore, using hydrophilic TDN, the phase transfer from the oil phase to the water phase was promoted, and due to the orderly distribution of TDN on the surface of the nano HAp, the monodispersity of the nanoprobe was also improved. Moreover, adding TDNs increased the stability of the targeted tumor, which showed that they had great potential in biomedical applications [63].

Bond et al. found that the Co^2+^ isomer (1,4,8,11-tetra(carbamoylmethyl)-1,4,8,11-tetraazacyclotetradecane, CCRM) produced four high CEST peaks with a paramagnetic shift. The 1,8-stranded pituitary of the complex was combined in a trans-arrangement to produce a Co^2+^ complex with higher kinetic inertia. Both 1,8 and 1,4-isomers had high paramagnetic shifts of the amide protons, and thus, they were effective pH probes whether in solution or in tissue homogenate [64].

The alanine residue on the oxidized collagen acts as a rich target of lung fibrosis, and thus, MRI containing small molecule Gd nanoprobe could specifically detect the stage of fibrosis in mouse models. Akam et al. proposed an upgraded nanoprobe GD-CHyD, containing N, N-dialkylhydrazine, which had higher reactivity and affinity for aldehydes. In experiments on mice with bleomycin-induced lung injury, the authors found that by using the increased reactivity and affinity of Gd-CHyD, the contrast between lung and liver could be improved. Therefore, the lung fiber molecular MRI nanoprobe could be improved by increasing the reactivity of hydrazine and the affinity for allylamine [65].

The fluorinated, thulium complex (Tm-PFZ-1) is used as an offline ^19^F-MRI nanoprobe for Zn^2+^. The combination of Tm^3+^ paramagnetic relaxation and chemical shift effect as well as the rapid exchange between different conformations could effectively eliminate the ^19^F-MRI signal in Tm-PFZ-1. The chelation of Zn^2+^ could increase structural rigidity and reduce the exchange rate, thereby providing a reliable ^19^F-MRI signal. Additionally, Tm-PFZ-1 provided an “off-on” response to Zn^2+^ in aqueous solution [12]. Kadakia designed an inorganic nanoprobe based on Ni^2+^ converted from low spin (S = 0) to high spin (S = 1). Light or β-galactosidase activated diamagnetic NiL(1) and NiL(2), respectively, and converted them into paramagnetic NiL(0), which increased the ^19^F-MRI relaxation rate. The ^19^F-MRI technology distinguishes chemical shifts and relaxation time to differentiate the signals. Thus, this nanoprobe could effectively detect light or enzyme expression in living cells [66]. The table summarizes the design of the environment-responsive MRI nanoprobes mentioned in the review, including the name of the probes and the resulting effect (Table 1).

## 3. The Application of the MRI Nanoprobe

Molecular nanoprobes have good targeting ability, high SNR, and deep tissue in situ imaging. They are important tools for tumor diagnosis and treatment. Since MRI technology has strong penetrating power and high spatial resolution, it is often used to monitor the drug transport process in organisms, combining MRI and molecular nanoprobes to design an environment-responsive nanoprobe that could remove background interference when imaging deep tissues in tumor detection and imaging. Bo et al. introduced amphiphilic fluorinated molecules into liposome bilayer vesicles, and encapsulated adriamycin in these liposome vesicles, which could be used to trace liposome drug delivery systems. The three monodisperse polyethylene glycol branches in the fluorinated molecule were used as hydrophilic ends to help improve the water solubility, stability, and biocompatibility of the nanoprobe. The fluorinated fragment was inserted into the bimolecular of the vesicle as the hydrophobic end to ensure the mobility of fluorine-containing fragments to provide better ^19^F-MRI signal [67]. Sekar et al. modified polyethylene glycol (PEG-FA) with doxorubicin (DOX), methoxy polyethylene glycol (mPEG), and folic acid, and combined with Fe_3_O_4_@Au to form a multifunctional nanomaterial (Fe_3_O_4_@Au-DOX-mPEG/PEG-FANPs) that could realize the dual role of tumor imaging and treatment [68]. The biotinylated thiosemicarbon dextran-coated iron oxide NPs, prepared by the researchers, had dual-function imaging capabilities. The aldehyde group of the oxidized dextran coating was used to couple biotin on the surface of NPs as a tumor targeting agent and thiosemicarbazide was used as the cationic chelating agent. It was compatible with the red blood cells but did not affect the clotting time. Compared with non-biotinylated cells, its advantage was also reflected in its significantly enhanced affinity for biotin receptor-positive 4T1 cells. In PET-MRI medical examinations, the early detection of biotin receptor-positive tumors could be achieved using radiolabeled NPs [69].

Wang et al. designed two types of tumor environment reaction branched and Gd-based glycopolymer conjugates. These branched macromolecules were prepared by one-pot reversible addition fragmentation chain transfer (RAFT) polymerization and conjugation chemistry. A biodegradable GFLG oligopeptide was used to successfully link the branched chains of the branched macromolecules, and finally a conjugate of the branched macromolecules and DOTA-GD (HB-pGAEMA-GD) was formed. The authors combined a tumor-targeting cRGDyK cyclic peptide to a branched molecule and prepared a tumor-targeting branched macromolecule-DOTA-GD conjugate (HB-pGAEMA-RGD-GD). This nanoprobe could significantly enhance the MRI signal intensity at the tumor site in vivo [70].

**Figure 3:**
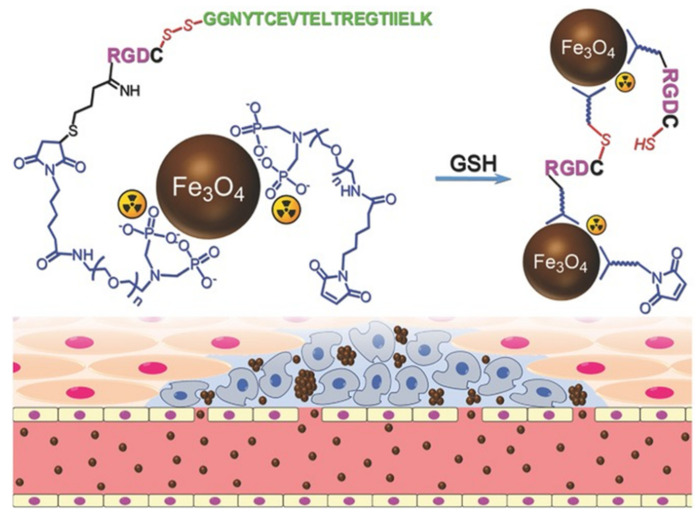
The design of ^99m^Tc-labeled anti-phagocytosis Fe_3_O_4_ nanoparticles and the cross-linking reaction between the particles to trigger the formation of particle aggregation in the tumor microenvironment by GSH [71]. Reproduced with permission from Wiley-VCH GmbH, Weinheim 2017.

Gao et al. modified Fe_3_O_4_ NPs using in situ cross-linking reactive peptide sequences to achieve enhanced tumor imaging (Figure 3). The tumor-specific Arg-Gly-Asp peptide and the self-peptide were connected by disulfide bonds. In the tumor environment, peptides were cleaved using glutathione, facilitating the reaction between the exposed thiol groups and the maleimide groups located in adjacent particles. The labeling responsive particle nanoprobes with ^99m^TC, combined with the aggregation of Fe_3_O_4_ NPs triggered by the tumor microenvironment resulted in the enhancement of the T_2_ effect, it had anti-phagocytic surface coating, active targeting ability, and dual-mode imaging [71].

Sepsis is one of the most common diseases in the world. It is a complex disease caused by the host’s poor response to infection. Without early treatment, it can lead to multiple organ failure and death. Nanotechnology is not only helpful for antibiotic therapy, but also for the early diagnosis of sepsis. Due to its unique characteristics and easy functionalization for specific pathogens, NPs have high diagnostic sensitivity and support multi-species detection [72]. Wang et al. designed a new type of activatable nanoprobes (ROS CAs), composed of clinically approved iron oxide cores, Gd-DTPA and hyaluronic acid (HA), which can be imaged to submicromolar concentrations by MRI, and they can be used as a sensitive contrast agent for sepsis assessment. The nanoprobe can undergo ROS-triggered degradation and release Gd-DTPA in the presence of ROS, resulting in the recovery of quenched T_1_-weighted MRI signals with rapid response. The results of in vitro and in vivo experiments show that ROS CAs can not only perform ROS imaging for early sepsis assessment, but also accurately track systemic ROS to assess the severity of sepsis [73].

**Figure 4:**
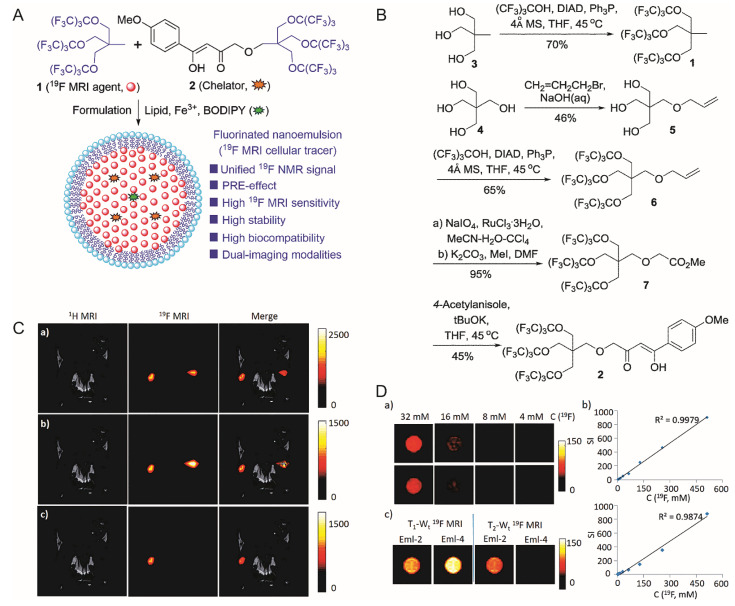
(**A**): Design of the nanoemulsion as ^19^F-MRI cellular tracer. (**B**): Synthesis of ^19^F-MRI agent 1 and chelator 2. (**C**): In vivo, ^19^F-MRI tracking of Eml-2- and Eml-4-labeled RAW264.7 cells in mice. (Upper) ^19^F density MRI; (Middle) T_1_-weighted ^19^F-MRI; (Lower) T_2_-weighted ^19^F-MRI; (Left) Eml-2-labeled cells were injected; (Right) Eml-4 labeled cells were injected for comparison. (**D**): (Upper left, middle left) ^19^F density MRI of Eml-2 and Eml-4, respectively; (Right) is SI versus C(^19^F) of Eml-2 and Eml-4, respectively; (Lower left) ^19^F T_1_/T_2_-W_t_ MRI of Eml-2 and Eml-4 at 9.4T. [76]. Reproduced with permission from Royal Society of Chemistry 2018.

Studies have shown that iron deposition is often found in the brains of patients who have neurodegenerative diseases. Ryo et al. utilized tetrafluoro-p-phenylenediamine (TFPDA) as a ^19^F signal transmitter, which had a Fe^2+^ selective chemical switch. Considering the N-oxide chemistry, it produced symmetry-dependent Fe^2+^ response to the change in the ^19^F signal. The addition of Fe^2+^ to F-Nox-1 triggered changes in the ^19^F signal chemical shift and signal intensity. The response to Fe^2+^ had a higher selectivity than other bio-related metal ions. Additionally, the designed nanoprobe had the ability to detect Fe^2+^ in serum by ^19^F-MRI, which also contained multiple biological pollutants. The ^19^F-MRI imaging of the soluble Fe^2+^ species facilitated the direct monitoring of the increase in Fe^2+^ levels before iron deposit formation, which was a potential risk factor for neurodegenerative diseases. MRI nanoprobes could achieve high-efficiency targeted recognition of Aβ polypeptide and phosphorylated Tau protein through chemical modification. They had good biocompatibility, were conducive to penetrating the blood–brain barrier (BBB), and could significantly enrich the nanoprobe in the lesion area. They had higher imaging sensitivity and specificity than traditional Alzheimer’s disease imaging methods and provided a strong support for the early diagnosis of AD [74,75]. Researchers also analyzed cell labeling and tracing, such as stem cells, lymphocytes, nerve cells, and macrophages. Peng et al. used a phospholipid monolayer to coat molecules containing 27 fluorine atoms, fluorine-containing chelating agents and BODIPY fluorescent dyes, obtaining a highly stable and highly biocompatible bimodal nanoprobe for cell labeling [76] (Figure 4).

DNA recognizes and pairs with complementary sequences through highly specific base-pair interactions. Kieger et al. hybridized and matched the DNA strands with 5-fluorouracil as the terminal group with complementary DNA sequences immobilized on the surface of Au NPs. The tight combination of fluorine-containing bases and the surface of Au NPs significantly weakened the ^19^F-MRI signal. In the presence of target DNA in the system, the fluorine-containing base DNA was released and the signal was restored [77]. Sicilia et al. designed a new type of ^19^F-DNA polymer-conjugated material composed of a linear methacrylamide backbone. This linear methacrylamide backbone was functionalized by single-stranded DNA and served as an anchor point to graft partially complementary fluorine-labeled DNA [78]. Pais et al. coupled the estrogen inhibitor 17β-estradiol and the estrogen activator Tamoxifen to PTA-Gd to prepare breast cancer estrogen receptor (ER)-based MRI molecules nanoprobes. The nanoprobes EPTA-Gd and TPTA-Gd, by imaging tumor mouse lesions, proved that EPTA-Gd had a targeted imaging effect in the lesions with ER-positive expression [79]. The table summarizes the application of the environment-responsive MRI nanoprobes mentioned in the review (Table 2).

## 4. Conclusions

MRI is undergoing a fast transformation from traditional non-specific physical imaging to specific molecular and gene-level imaging. Disease evaluation indicators are also changing from traditional morphological changes and anatomical positioning to changes in enzyme function, receptor levels, and gene expression. MR molecular imaging is known to provide a more comprehensive, 3D, specific imaging and biological data for diseases, and shows broad application prospects in life sciences, basic medicine, and clinical research. The diseased tissue microenvironment is significantly different from normal tissues. Thus, using the physical and chemical properties of the diseased tissues to develop environment-responsive MRI nanoprobes can not only improve the imaging effect of the tumors, but also assist in disease treatment. From an imaging point of view, achieving the clinical transformation of environment-responsive nanoprobes requires improving the sensitivity of MRI. Thus, the current strategy aims to obtain a good signal-to-noise ratio with a longer scan time. Future studies might focus on improving material design to improve sensitivity. MR molecular imaging would further facilitate the diagnosis and treatment of diseases; however, we should also strive to improve the deficiencies that still exist in MR molecular imaging, such as nanoprobe safety, imaging sensitivity, etc.

## Figures and Tables

**Table 1:** Summary of the design of environment-responsive MRI nanoprobes.

	Probe	Consequence and Effect	Ref.
pH-responsive MRI nanoprobes	PEGMnCaP	It dissolved at an acidic pH, and the released Mn^2+^ could combine with the proteins, increasing the T_1_ contrast.	[26]
	ATO@SiO_2_ NPs	The acidic medium triggered the simultaneous release of the clinical anticancer drug ATO and Mn^2+^, enhancing the contrast of T_1_.	[27]
	MnO NPs @SiO_2_	In an acidic environment, Mn^2+^ was released, enhancing T_1_ contrast.	[28]
	MnOx-HMCNs	A mildly acidic solution could increase its T_1_ relaxation value by 52.5 times. It showed an anti-metastatic effect and high performance in reversing cancer cell multi-drug resistance.	[30]
	Fe_3_O_4_ NPs @Micelles	At pH < 6.8, the micelle ruptured, releasing the iron oxide NPs and enhancing the T_2_ contrast.	[31]
	^19^F-Peptide nanostructures	In an acidic environment, due to the increased mobility of fluorine probes in cylindrical nanostructures, their arrangement was cylindrical, turning the ^19^F-MRI signal “on”.	[32]
	Au NPs @mesoporous SiO_2_ NPs	At pH < 6, the hydrazine bond was hydrolyzed and the fluorine nanoprobe was released, consequently activating the ^19^F-MRI signal.	[33]
	^19^F-Micelles	By decomposing the micelles, it could achieve pH-based environmental response and qualitative measurement of the environmental pH values by responding to ^19^F-MRI.	[34]
	GdNP-DO3A	The nitrophenol group was protonated at low pH, allowing water to approach Gd. An increase in pH caused an increase in the relaxation performance.	[35]
	Au@Gd&RGD	It could be used to monitor pH changes of lysosomes in living cells due to its sensitivity to acidic conditions.	[36]
	D-Au@Gd&RGD	It could obtain a precise intracellular pH map and quantitatively calculate the pH values of living cells.	[36]
	GMF&drug molecules @NPs	Under acidic conditions, the hydrophobic-hydrophilic transition of the pH-responsive polymer caused the amplification of the MRI signal, resulting in the rapid release of the drug.	[37]
Enzyme-responsive MRI nanoprobes	C-SNAM	In the reducing environment of GSH in the cell, cyclization was triggered by the degradation of DEVD peptide in the presence of caspase 3/7. The amplification of r_1_ in Gd NPs and the tissue retention due to the increase in size caused T_1_ contrast enhancement in MRI.	[38]
	Gd chelate-^19^F	When the peptide was cleaved by caspase 3/7, the Gd chelate was separated from fluorine, and the ^19^F-MRI signal was turned on.	[40,41]
	fluorinated hydrogel precursor	Tyrosine kinase controlled the decomposition of the hydrogel and subsequent turning-on of the ^19^F-MRI signal.	[44]
	Gd-peptide	When the nanoprobe interacted with PDI, the nanoprobe bound to fibrin, increasing r_1_ by 70%.	[45]
	Gadoteridol@liposomes	In the presence of PLA_2_, liposomes were degraded and Gd probes were released, leading to a significant reduction in T_1_ relaxation time.	[46]
	IO NPs (MMP-9)	After MMP-9 sheared the IO, it released the PEG molecule, enhancing the T_2_ relaxation effect.	[47]
	IO NPs (MMP-14)	At the tumor site, MMP-14 cleavage of the peptide, resulting in the accumulation of nanoprobes in the tumor and enhancing the T_2_ contrast.	[48]
	Salicylic acid derivative	Sulfatase and esterase cleaved the probe, turning the CEST signal “on”	[50]
Redox-responsive MRI nanoprobes	Fe_3_O_4_@Mn_3_O_4_	In the presence of GSH, the shell decomposed into Mn^2+^ exposing iron oxide NPs and increasing r_1_ and r_2_.	[55]
	^19^F-Fe^3+^ chelate	When APS oxidized Fe^2+^ to Fe^3+^, the signal was turned “off”. A mild reducing agent could reduce the system to Fe^2+^ turning the signal “on” again.	[56]
	Gd chelate–SiO_2_ NPs	The presence of GSH could separate Gd chelate from SiO_2_, significantly increasing r1.	[57]
	^19^F@SiO_2_-Gd-chelate	The reducing environment could not only break the disulfide bond but also separate the Gd chelate from the fluorine probe, thereby turning the ^19^F-MRI signal “on”.	[58]
	CuL_1_ and CuL_2_	They retained their initial quenched ^19^F-MRI signal. When the complex was reduced, the signal increased.	[59]
	Cu^2+^ ATSM derivatives	Adjusting the distance between Cu^2+^ and F atoms could enhance ^19^F-MRI relaxation.	[11]
	Branched fluorinated glycoprotein	In a reducing environment, the polymer exhibited an enhanced ^19^F-MRI signal.	[61]
Other examples of MRI nanoprobes	Ce6/Fe_3_O_4_-M	The elemental oxygen generated by light irradiation triggered the cleavage of TK, obtaining a negatively enhanced T_2_-weighted MRI signal.	[62]
	Apt-TDNs-GdHAp	TDNs enhanced the monodispersity of the nanoprobe and improved the stability and accessibility of targeted tumors.	[63]
	CCRM	1,8 and 1,4-isomers had paramagnetically shifted amide protons, which acted as excellent pH probe.	[64]
	GD-CHyD	The increased reactivity and affinity of Gd-CHyD could improve the contrast between the lung and the liver.	[65]
	Tm-PFZ-1	Tm^3+^ could eliminate the ^19^F-MRI signal; chelation of Zn^2+^ could provide ^19^F-MRI signal.	[12]
	inorganic probe-Ni^2+^	It increased the ^19^F-MRI relaxation rate. This nanoprobe could detect light or enzyme expression in living cells.	[66]

**Table 2:** Summary of the application of environment responsive MRI nanoprobes.

Probe	Application	Ref.
adriamycin@vesicles	They can be used to trace liposome drug delivery systems and ensure the mobility of fluorine containing fragments and provide better ^19^F-MRI signals.	[67]
Fe_3_O_4_@Au-DOX-mPEG/PEG-FANPs	They can realize the dual role of tumor imaging and treatment.	[68]
HB-pGAEMA-RGD-GD	The nanoprobe has been shown to significantly enhance the MRI signal intensity at the tumor site in vivo.	[69]
Arg-Gly-Asp Fe_3_O_4_ NPs	They can enhance the T_2_ effect and possess anti-phagocytic surface coating, active targeting ability, and dual-mode imaging.	[71]
TFPDA	They have higher imaging sensitivity and specificity, and provide strong support for the early diagnosis of AD.	[74,75]
phospholipid coat molecules	They can prepare highly stable and highly biocompatible bimodal nanoprobes for cell labeling.	[76]
5-fluorouracil &Au NPs	In the presence of target DNA in the system, the fluorine-containing base DNA is released, restoring the signal.	[77]
^19^F-DNA polymer	They serve as an anchor point to graft partially complementary fluorine-labeled DNA.	[78]
ER molecules probes	They have a targeted imaging effect in the lesions with ER-positive expression.	[79]
